# AAF-Net: Scene text detection based on attention aggregation features

**DOI:** 10.1371/journal.pone.0272322

**Published:** 2022-08-05

**Authors:** Mengmeng Chen, Mayire Ibrayim, Askar Hamdulla

**Affiliations:** 1 College of Information Science and Engineering, Xinjiang University, Urumqi, China; 2 Xinjiang Key Laboratory of Signal Detection and Processing, Urumqi, Xinjiang, China; University of Engineering & Technology, Taxila, PAKISTAN

## Abstract

With the advent of the era of artificial intelligence, text detection is widely used in the real world. In text detection, due to the limitation of the receptive field of the neural network, most existing scene text detection methods cannot accurately detect small target text instances in any direction, and the detection rate of mutually adhering text instances is low, which is prone to false detection. To tackle such difficulties, in this paper, we propose a new feature pyramid network for scene text detection, Cross-Scale Attention Aggregation Feature Pyramid Network (CSAA-FPN). Specifically, we use a Attention Aggregation Feature Module (AAFM) to enhance features, which not only solves the problem of weak features and small receptive fields extracted by lightweight networks but also better handles multi-scale information and accurately separate adjacent text instances. An attention module CBAM is introduced to focus on effective information so that the output feature layer has richer and more accurate information. Furthermore, we design an Adaptive Fusion Module (AFM), which weights the output features and pays attention to the pixel information to further refine the features. Experiments conducted on CTW1500, Total-Text, ICDAR2015, and MSRA-TD500 have demonstrated the superiority of this model.

## Introduction

Scene text detection is an important research direction in the field of object detection, with the goal of detecting text in real-world photographs. Recently, with the upsurge of deep learning, scene text detection has been widely applied in driverless, unmanned supermarkets, image retrieval, real-time translation, and other fields.

Compared with general object detection, scene text has various shapes and sizes, variable text language, different text styles, and is easily affected by lighting, so it faces huge challenges. Previous scene text detection methods [1–6] mainly rely on manually set features to understand images. The current rise of deep learning mainly divides this task into regression-based and segmentation-based methods. Although the regression-based approach has a fast detection speed, it does not perform well in special-shaped text datasets such as vast aspect ratio, curved text, etc. Segmentation-based methods have been improved on curved text datasets. However, most of these methods suffer from large models and slow detection speeds. Moreover, these methods are difficult to detect small text and separate adjacent text instances. Generally speaking, the detection of arbitrary-shaped text in complex scenes currently faces the following problems: First, high-precision models have a large number of parameters and are time-consuming, making it difficult to apply in practical industries. Second, the scale of text instances in scene text varies widely. The current scene text algorithm can accurately detect large-scale text, but it is difficult to detect small-scale text, and it cannot separate adjacent text in dense areas. The lightweight detector Pixel Aggregation Network (PAN) algorithm proposed by Wang et al. [7] uses ResNet-18 as the backbone, uses a Feature Pyramid Enhancement Module (FPEM) and Feature Fusion Module (FFM) to solve the lightweight network detection problem, and aggregates text pixels by predicting similarity vectors, which largely solves the speed problem of detecting arbitrarily oriented text. However, the network detects text objects of different scales in images of specific scales, each feature map in the pyramid (used to detect a specific size object within range) mainly or only consist of a single level of features. In the path of feature enhancement, since the linear interpolation method used in the up-sampling process only depends on the relative position, and the element addition method is used to fuse two adjacent features after up-sampling, which ignores the semantic difference between feature maps due to different depths. Second, the representational power of high-level semantic features is not fully utilized in larger receptive fields. For the feature fusion module, a simple up-sampling and connection method is used for feature fusion. However, there are semantic differences in cross-scale feature maps, so up-sampling may result in the problem that the ratio of the receptive field of the feature map and the text feature does not match, and the information cannot be fully fused. From the analysis of the experimental results, this method produced serious misdetection and omission for small-scale text or separation of adjacent text. PAN++ [8] is an end-to-end scene text detection and recognition network. Its detection network is slightly improved in PAN [7], which adds the feature combination of the corresponding scale in the FFM module directly to the scale enhancement path. In other words, the output feature pyramid is the outcome of combining the pieces of the input feature pyramid with the scale-enhanced feature pyramid.

Inspired by the above analysis, in this paper, we continue to use lightweight ResNet-18 as the backbone for feature extraction, but we employ a more effective method to enhance features, the Cross-Scale Attention Aggregation Feature Pyramid Network (CSAA-FPN). The Feature Attention Aggregation Module (AAFM) in the network effectively solves the multi-scale variation problem in scene text by fusing adjacent features and skipping connections. Specifically, we use the concatenate operator to fuse two neighboring features, aggregating feature complementary information and making full use of semantic information at different levels. Secondly, we utilize contextual information to improve the feature representation, and by directly adding convolutional layers to expand the receptive field while preventing increased computational burden. Finally, we use channel and spatial attention to enhance semantic consistency before outputting features, and further refine features from each feature level in the pyramid to make full use of channel and spatial information to solve the problem of feature level imbalance for text instances at different scales. Besides, we design weights to the fused features through an Adaptive Fusion Module (AFM), which they will focus on accurate features while abandoning inaccurate ones, and the classified features are used in the subsequent pixel aggregation post-processing algorithm.

The experiments on four challenging benchmark datasets including CTW1500, Total-Text, MSRA-TD500, and ICDAR2015 have demonstrated that the two proposed strategies can effectively enhance the detection performance. Specifically, they bring 4.9%performance improvement on multilingual text dataset MSRA-TD500 and 2.79% performance improvement on the curved text dataset CTW1500 dataset. Meanwhile, our model also has promising performance on multi-oriented and long text datasets.

In summary, the contribution of this paper is as follows: (1) A new U-shaped feature pyramid network for cross-scale attention aggregation features is proposed, guiding low-level semantic information to fuse multi-scale information layer by layer through high-level semantic information, performing top-down semantic feature enhancement and low-up detail information enhancement, and expanding features using jump connections. Furthermore, we apply CBAM attention to spatial and channel information to obtain more accurate resolution features as well as richer information in the output features. Compared with the original network, the network can detect small-scale text and adjacent text more effectively. (2) An adaptive fusion lightweight module is proposed to weight the output feature maps and focus on the location information of the features. This module can suppress useless feature information and effectively utilize pixel information to achieve feature selection. (3) The network of this paper is verified on different datasets. Compared to base-line models, the advanced performance is achieved without the use of pre-training finetuning.

## Related work

### Text detection algorithm

The background of the early images is relatively simple, and the form of the text also is relatively simple. The traditional methods [1–6] generally use the features designed by the human hand to detect the text and have achieved some good results. For example, Kim et al. [1] proposed a texture classifier characterized by the texture of the text, Guo et al. [2] proposed a detector using a sliding window, Epshtein et al. [3] proposed the stroke width algorithm, and the MSER algorithm [9], etc. Nowadays, under the condition of complex background, the algorithms based on deep learning are mainly divided into algorithms based on region suggestion and algorithms based on segmentation.

The region-based suggestion approach mainly uses the target detection frame-works of Fast R-CNN, SSD, and YOLO as the basic framework and improves it with practical applications. In the Deep Text algorithm proposed by Zhong et al. [10], Faster R-CNN was applied to the scene text detection algorithm for the first time, adding fuzzy text category information and a multi-scale region pooling module to better detect small-scale text. Although Faster R-CNN can achieve text localization, it is also only adapted to horizontal text. To address this difficulty, the RRCNN framework proposed by Ma et al. [11] adds rotation factors to the detection network to detect text in any direction. Zhu et al. [12] also uses the Faster R-CNN framework to improve the detection of curved text. Since the text itself generally exists as long rectangular text, the text is usually displayed in the image at different scales. In response to this feature, Shi et al. [13] proposed building a segmented SegLink network with the SSD infrastructure. TextBoxes [14] modifies the convolution kernel size of SSD to fit the more elongated text. RRD [15] further improves the detection rate in the long and multi-directional text by extracting different feature maps for regression and classification. Inspired by the YOLO network, Gupta et al. [16] proposed the FCRN model, which implements regression prediction for text-boxes based on a fully convolutional network. ContourNet [17] incorporates an adaptive RPN into the network to detect arbitrary shaped text. DDR [18] and EAST [19] use corner localization methods to predict text boxes. Although regression-based methods have achieved text detection, they mostly rely on bounding box settings, so it is not easy to achieve accurate results on extreme aspect ratios and curved text.

The segmentation-based method generally obtains text pixels by segmentation and obtains the text box position through pixel point regression or aggregating text pixels, which greatly increases the detector’s detection rate for arbitrarily shaped text. This type of method mainly detects text based on segmentation networks such as Mask R-CNN, FCN, and FCIS. SPC Net [20] improves on Mask R-CNN by using two different branches for text segmentation. PMTD [21] introduces location information for quadrilateral detection and obtains accurate text boxes through a planar clustering method. Mask Textspotter V1 [22] is an end-to-end detection recognizer that simultaneously performs both classification regression and segmentation of text, generating text instance segmentation maps and character segmentation maps. TextSnake [23] represents the text instance as a disc with information, predicts the text centerline and text area score map through the FPN network, and extracts the shape direction of the text instance to achieve accurate segmentation of the text instance. Zhu et al. [24] proposed the TextMountain algorithm for semantic segmentation of images, where the text is determined by pixel shifting. Pinxlink [25] is to view the text detection problem as an instance segmentation problem and to separate textual and non-textual regions by means of linking. Yang et al. [26] proposed IncepText which uses different convolutional kernels composed of different branches to detect different scales of text, while invoking deformable convolutional layers and deformable pooling layers for multi-directional text detection. Baek et al. [27] proposed that the CRAFT algorithm takes character segmentation as the core idea. The algorithm is based on the idea of weakly supervised learning, and after getting the prediction results, the Gaussian heatmap is used to generate the character label, and finally, the MSER algorithm is used to segment the character area. PSENet [28] performs text instance segmentation by using different scale kernel expansion methods.

### Feature pyramid network

FPN [29] constructed an effective framework to solve the multi-scale feature extraction problem by a top-down path and lateral connections for layer-by-layer feature fusion. This has been widely applied and is being further investigated. However, FPN suffers from two widespread limitations: (1) the information in the fusion process is constantly attenuated; (2) the semantic inconsistency between adjacent features may cause aliasing effects. AugFPN [30] improves FPN by proposing adaptive channel fusion, residual feature enhancement, and soft ROI selection modules to extract and fuse features, respectively. Networks such as FPN [29] and AugFPN [30] only use a single top-down path and horizontal connections for feature enhancement and fusion. It transfers high-level strong semantic features to the bottom layer to improve the expressiveness of the entire pyramid. However, low-level positional information from the bottom of the pyramid cannot be transferred to the top. This can lead to inaccurate localization of text bounding boxes, thus reducing detection performance. PAFPN [31] introduces bottom-up paths to further expand and make full use of the information in each feature layer. Networks such as PAFPN [31] and BiFPN [32] can alleviate these problems to a certain extent, but there is still room for further improvement. A^2^FPN [33] improves multi-scale features by adding attention to feature extraction and fusion, respectively.

Inspired by the appeal method, this paper proposes a cascaded U-shaped pyramid network by combining top-down and low-up paths, focusing on feature enhancement while reducing information loss due to channel decline and optimizing the final features after complex integration.

## Methodology

### Overall architecture

**Fig 1:**
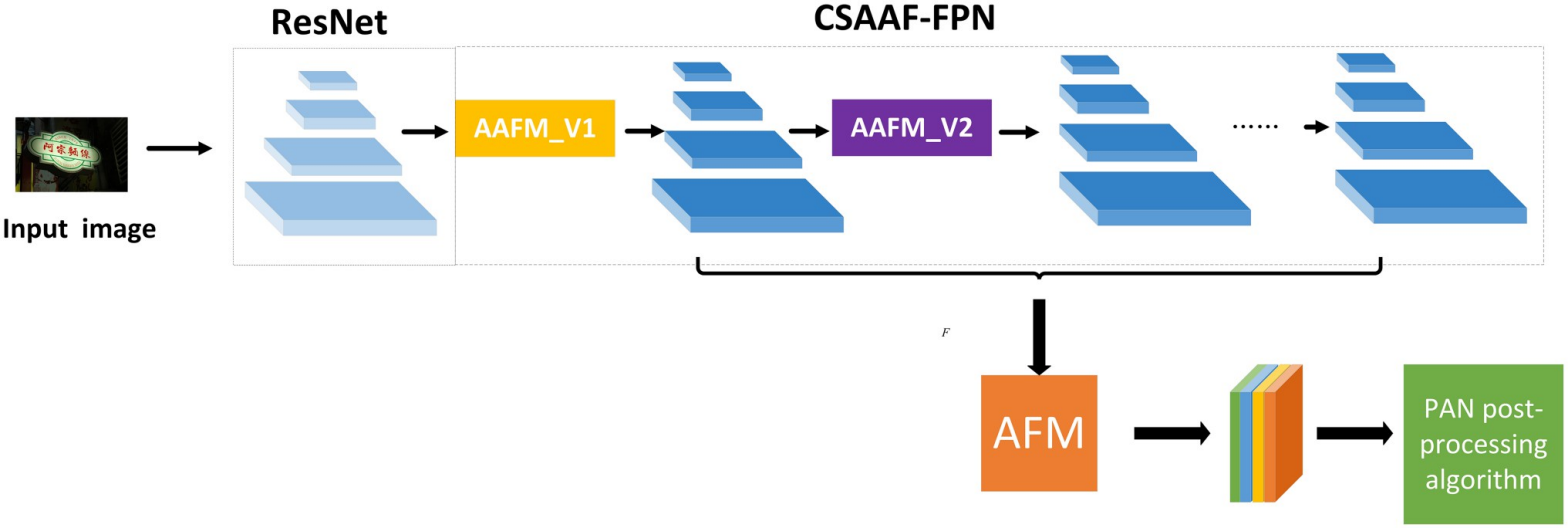
Overall architecture of AAF-Net. It includes a basic structure with ResNet-18; a cross-scale attention aggregation feature pyramid network (CSAA-FPN); an adaptive fusion module (AFM); the pixel aggregation algorithm. The features from lightweight backbone network are enhanced by a segmentation head which is composed of CSAA-FPN and AFM.

In recent years, the pyramid enhancement network has made significant breakthroughs in the image field. This paper takes the PAN [7] algorithm as the baseline and improves the original algorithm. The proposed algorithm is mainly divided into a cross-scale attention aggregation module and an adaptive fusion module. (Fig 1) shows the scene text detection structure in this paper. By improving the feature enhancement module, the information of adjacent layers is better utilized without adjusting the model infrastructure.

### Cross-scale attention aggregation feature pyramid network

**Fig 2:**
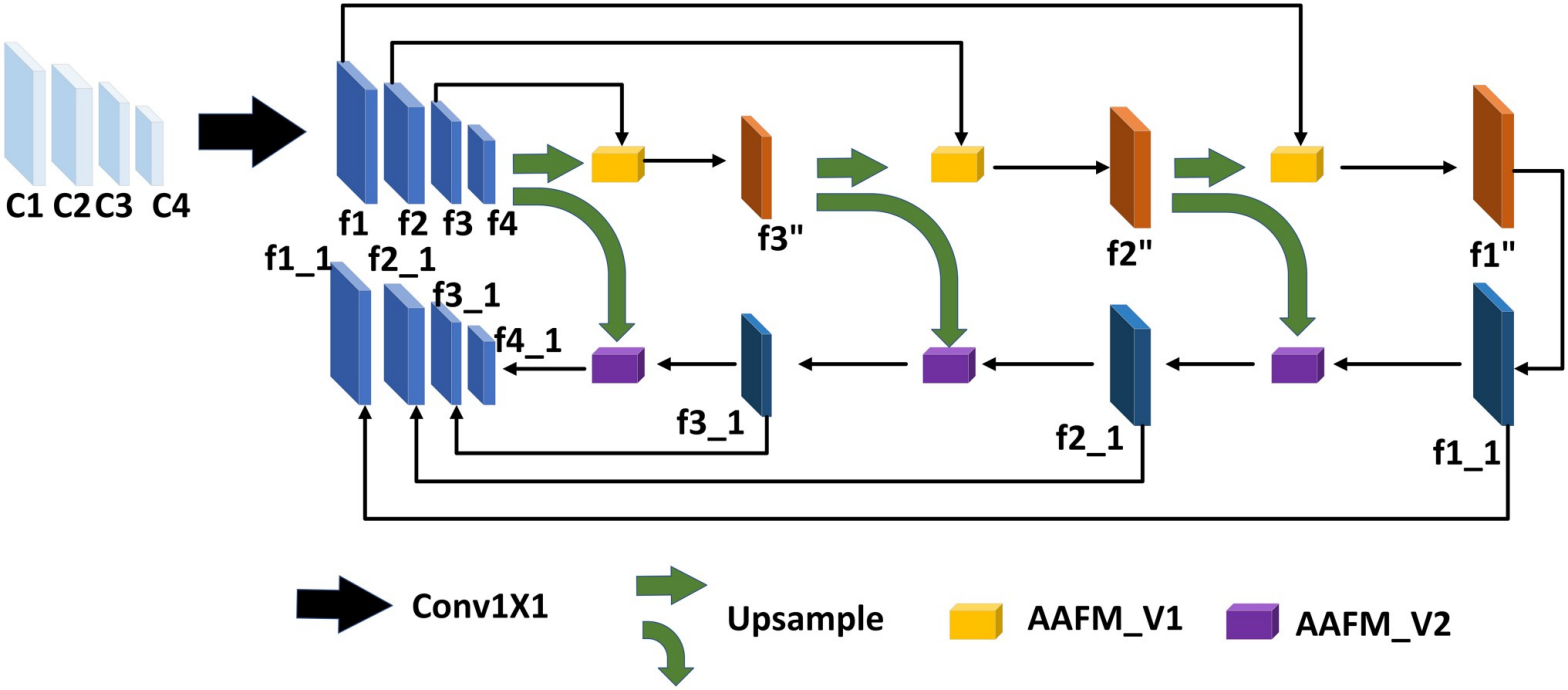
The proposed cross-scale attention aggregation FPN network framework.

There are objects of different sizes in different images, and each object has different feature information. Shallow information can distinguish simple features, while deep information can distinguish complex objects. There is often rich semantic information in the deep network but a lack of geometric information. On the contrary, there is rich geometric information in the shallow network but a lack of semantic information. Based on the structure of Feature Pyramid Enhancement Module, in this paper, we propose a Cross-Scale Attention Aggregation Feature Pyramid Network. As for the small object, the text has less pixel information and is easily lost in the up-sampling process, so the attention module is added to enhance the pixel information. The proposed architecture is shown in Fig (2).

CSAA-FPN is a U-shaped network architecture with path aggregation. Specifically, the feature information is augmented by the aggregation of lower-level information guided by the higher-level information. It includes two paths, a top-down high-scale enhancement through the AAFM_V1 module and a low-scale enhancement from low to high through the AAFM_V2 module, with a bottom-up lateral connection being the final output of the module, and we are using two cascaded networks to enhance the output features. The input{C1, C2, C3, C4} are the outputs of ResNet’s Conv2, Conv3, Conv4, and Conv5 respectively, and their size is $\left\{\frac{\text{1}}{\text{4}},\frac{\text{1}}{\text{8}},\frac{\text{1}}{\text{16}},\frac{\text{1}}{\text{32}}\right\}$ of the original image. Firstly, reduce the number of channels of C1, C2, C3, and C4 to 128 to obtain the feature layers f1, f2, f3, and f4 after dimension reduction. Secondly, a top-down path generates higher-resolution features, while a bottom-up path conveys geometric information to the top layer to enhance semantic and spatial information.

**Fig 3:**
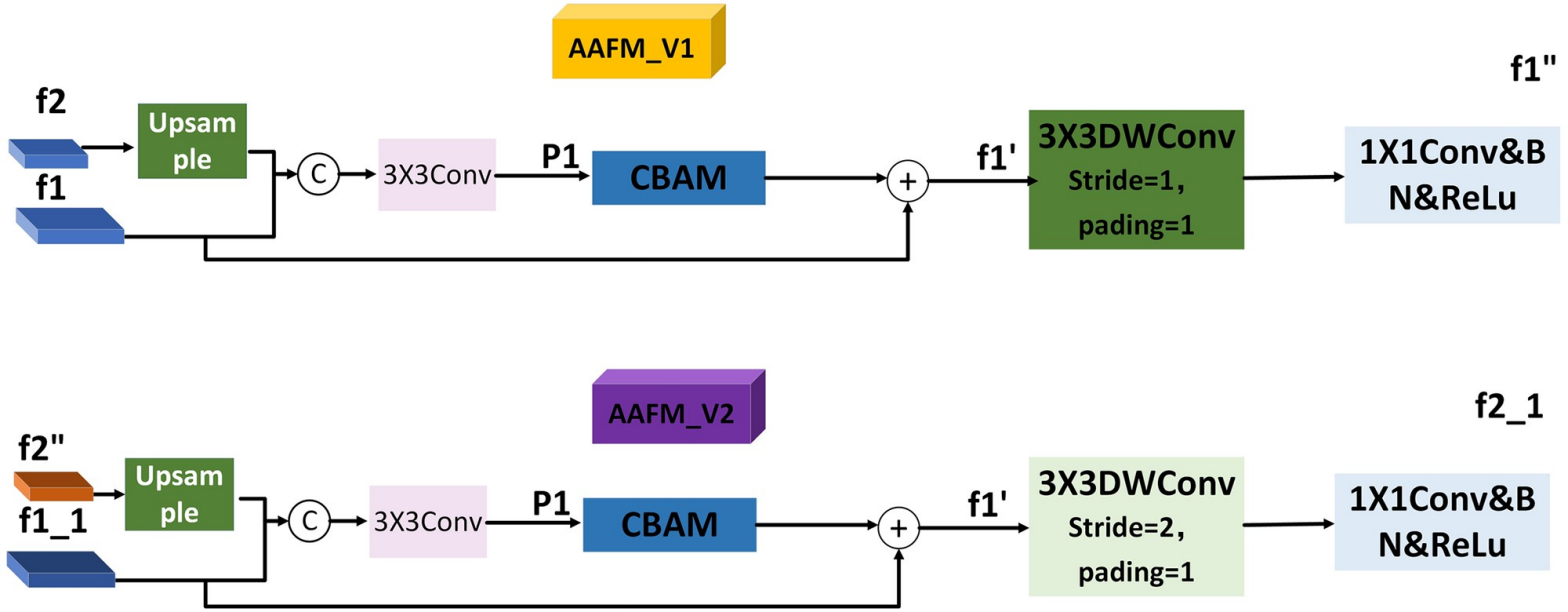
Attention aggregation feature module AAFM_V1, AAFM_V2.

We take f2 and f1 as an example. As shown in Fig (3), in the top-down attention aggregation feature module AAFM_V1. Firstly, a guided fusion of horizontal connections is performed, and the small-resolution feature f2 is sampled to the same scale as the large-resolution feature f1 by the bilinear difference method, and then connected.

$$\begin{array}{ccc} P1 & = & \text{Conv}(\text{cat}(f2↑,f1)) \end{array}$$ (1)

Where ↑ stands for up-sampling, cat[⋅] represents the concatenation operation, and Conv stands for 3 × 3 convolutional layers. The purpose of using concatenation is that the higher-level feature maps are rich in semantic information. The connection between the high-level and low-level features allows the rich semantic information to be transferred to the low-level features, so we connect this. In this way, feature information is enhanced by fusing high-level semantic features. Next, the generated features are computed by element-by-element convolution, and the intermediate features P1 are output. The 3 × 3 convolution increases the receptive field of the connected features and increases the local context information. P1 at each fusion level contains both semantic and detailed information, which can well preserve more discriminative and complementary representations on the progressive fusion path.

Second, we feed the output feature P1 into the attention module CBAM [34]. Since the up-sampling process causes the location of the deep features to change while causing the loss of information, the attention module CBAM is introduced to enhance the location and spatial information after the connection to make full use of the valuable information. Then, f1 is superimposed with the emphasized information through the correction branch so that informative features are enhanced, and fewer valuable features are suppressed to obtain the output feature map 1′.

$$\begin{array}{ccc} f1^{\prime } & = & f1+\text{CBAM}(P1) \end{array}$$ (2)

CBAM attention module is a lightweight module that mainly contains two attention modules: a network combining spatial attention mechanism and channel attention mechanism. It is a cascade of two modules, which are first passed through the channel attention module and then input to the spatial attention module to get the final information. The network learns to focus on the critical information more.

**Fig 4:**
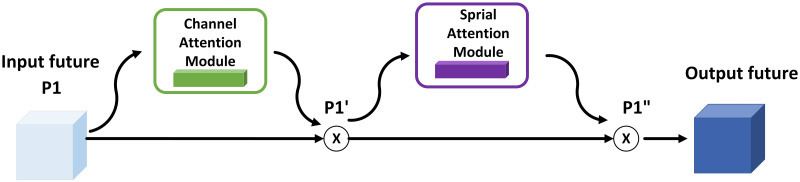
The structural framework of CBAM.

**Fig 5:**
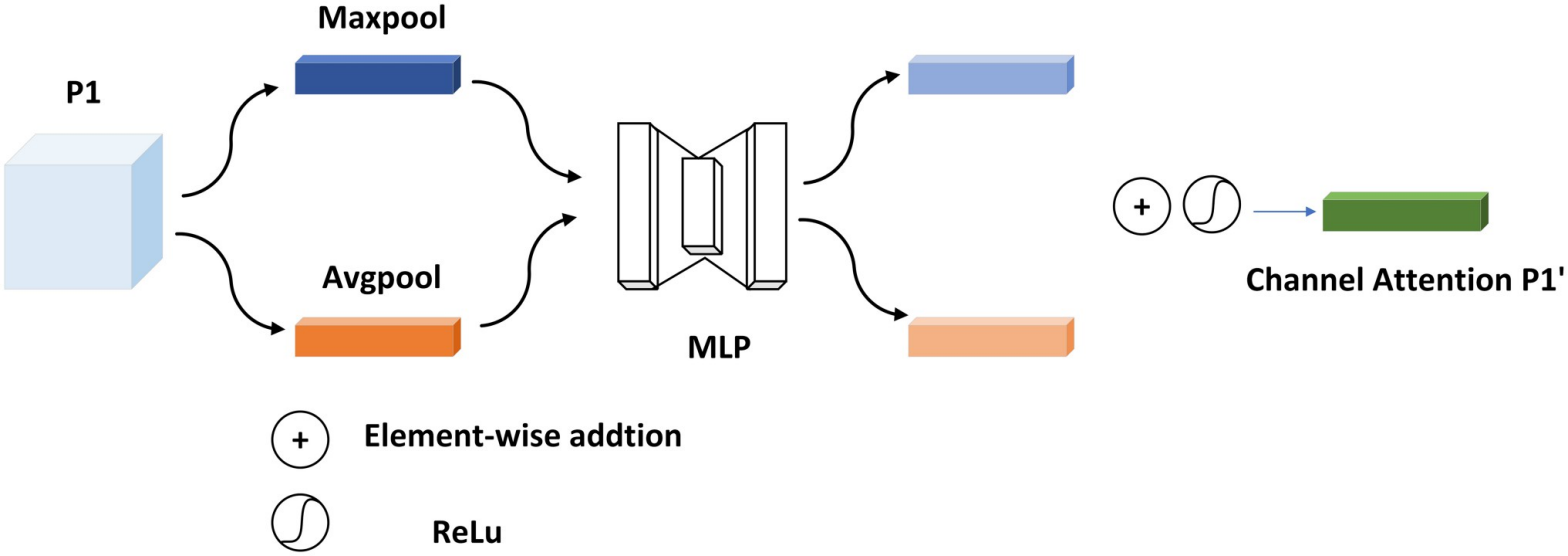
Channel attention module structure.

The structure of CBAM is shown in Fig (4):

Among them, the channel attention module pays attention to the critical information on the channel. As shown in Fig (5), the input feature P1 first obtains the global channel information through the global average pooling layer and the global max-pooling layer based on the spatial size, respectively. The global average pooling gets the average value of feature maps for each channel, and the max global pooling gets the maximum value of each channel’s features. Then it is fed into a two-layer neural network MLP with shared features, and the two output features are summed pixel by pixel. Finally, the channel attention feature maps are obtained by activating the features with an activation function Sigmoid.

$$\begin{array}{ccc} P1^{\prime } & = & \sigma (\text{MLP}(\text{AvgPool}(P1))+\text{MLP}(\text{Maxpool}(P1))) \end{array}$$ (3)

$$\begin{array}{cc} = & \sigma (W_{1}(\;W_{0}(P_{\text{avg}}^{c})+W_{1}(\;W_{0}(P_{\max}^{c}))) \end{array}$$ (4)

Where *σ*(⋅) is the activation function and W_0_ is the weight of the first-layer MLP, and W_1_ is the weight of the second-layer MLP.

**Fig 6:**
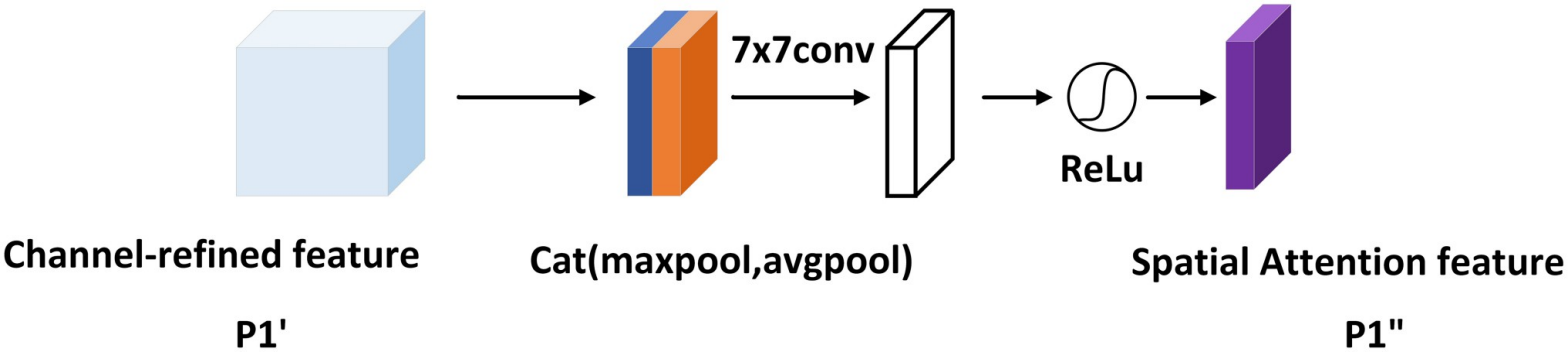
Spatial attention module structure.

In Fig (6), the channel attention feature map P1′ is multiplied pixel-by-pixel with the input features as the input to the spatial attention module, which focuses on spatial information. Similarly, the spatial attention module obtains global spatial information through the channel-based global average pooling layer and the global maximum pooling layer, respectively. The difference is that the two feature maps’ output afterward is channel-connected, downscaled by a 7 × 7 convolution, and the perceptual field increases. Then, the activation function activates the features to obtain the features P1′′ after spatial attention and channel attention.

$$\begin{array}{ccc} P^{\prime \prime } & = & \sigma (\text{Conv}7\times 7(\text{cat}(\text{AvgPool}(P1^{\prime }),\text{Maxpool}(P1^{\prime })))) \end{array}$$ (5)

$$\begin{array}{cc} = & \sigma (\text{Conv}7\times 7(\text{cat}(P_{\text{avg}}^{s},P_{\max}^{s}))) \end{array}$$ (6)

Finally, the obtained feature map is subjected to a 3 × 3 depthwise convolution and a 1 × 1 point-by-point convolution with a stride of 1 in the top-down path, and then activated. BN is normalization and ReLu is the activation function.

$$\begin{array}{ccc} f1^{\prime \prime } & = & \text{ReLu}(\text{BN}(\text{Conv}1(\text{Conv}3(f1^{\prime }))) \end{array}$$ (7)

In the top-down process, f3, f2, f1 are enhanced in turn to obtain f3′′, f2′′, f1′′, and similarly, f4, f3′′, f2′′, f1′′ are augmented in the low-up path to obtain f1_1, f2_1, f3_1, f4_1. In the case of the low-up path, the 3 × 3 depthwise convolution with a step size of 1 becomes a 3 × 3 depthwise convolution with a step size of 2 for the purpose of scale alignment. The purpose of 3 × 3 depthwise convolution is to expand the receptive field, while the 1 × 1 point-by-point convolution is used to deepen the depth and improve the network’s learning ability, thereby greatly improving the fusion effect and achieving better performance.

$$\begin{array}{ccc} fi^{\prime \prime } & = & \left\{ \begin{array}{c} f_{4},i=4\\ \text{Relu}(BN(f^{1\times 1}(f^{3\times 3(1)}(fi^{\prime }))),i=1,2,3 \end{array}\right. \end{array}$$ (8)

$$\begin{array}{ccc} fi_{-}1 & = & \left\{ \begin{array}{c} f_{1}^{\prime \prime },i=1\\ \text{Relu}(\text{BN}(f^{1\times 1}(f^{3\times 3(2)}(fi^{\prime \prime })))),i=2,3,4 \end{array}\right. \end{array}$$ (9)

### Adaptive fusion module

**Fig 7:**
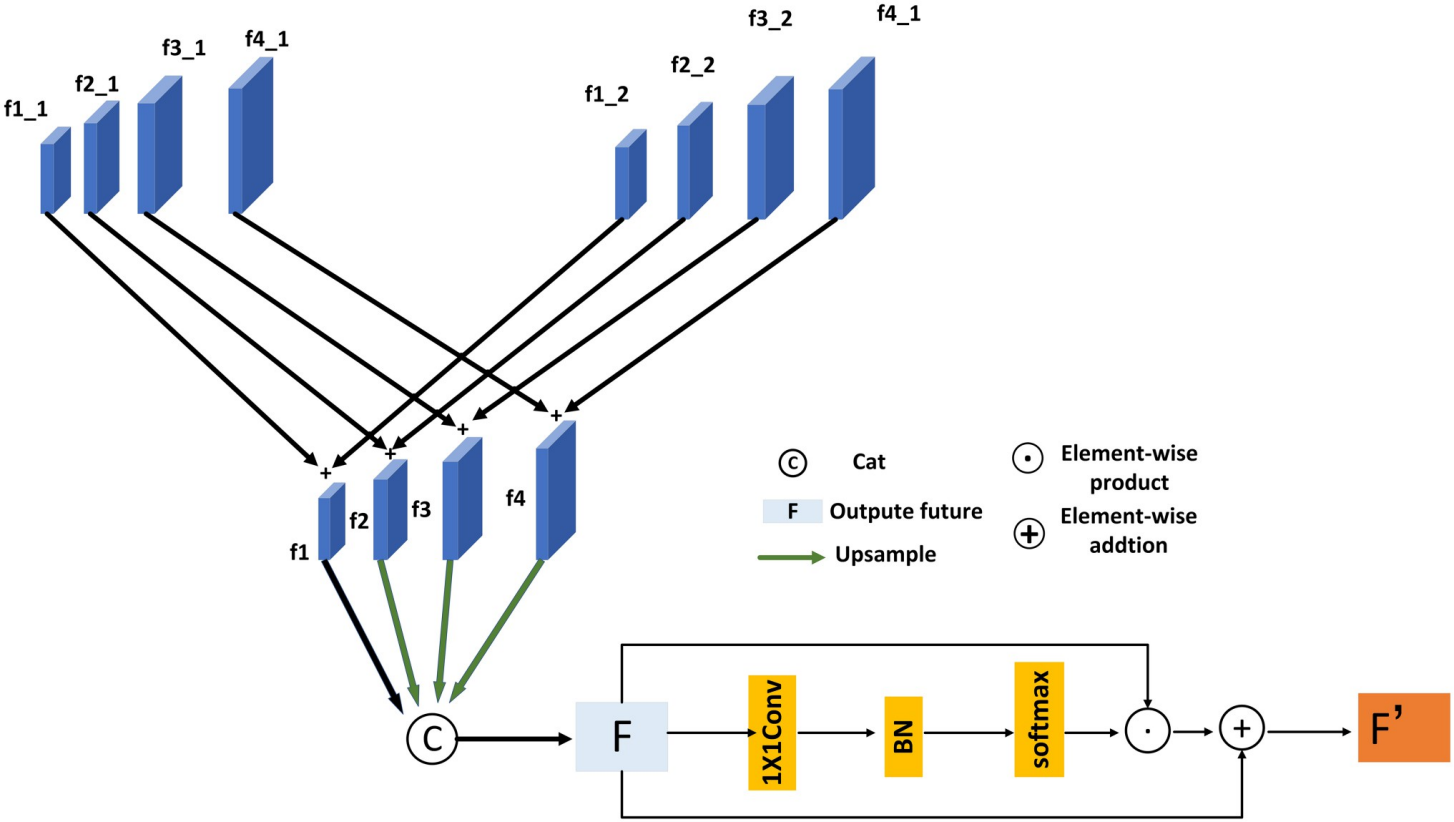
Adaptive fusion module structure.

In this paper, we propose a cross-scale attention aggregation FPN network to enhance the feature information, and subsequently in the output feature stage we need to fuse the enhanced information and output the overall features. Thus, we propose an adaptive fusion module as shown in Fig (7).

We firstly combine the corresponding scale feature maps by element addition, the integration of the corresponding scale features can be expressed as:

$$\begin{array}{ccc} f1 & = & f_{1_{-}1}+f_{1_{-}2} \end{array}$$ (10)

$$\begin{array}{ccc} f2 & = & f_{2_{-}1}+f_{2_{-}2} \end{array}$$ (11)

$$\begin{array}{ccc} f2 & = & f_{3_{-}1}+f_{3_{-}2} \end{array}$$ (12)

$$\begin{array}{ccc} f4 & = & f_{4_{-}1}+f_{4_{-}2} \end{array}$$ (13)

And in the process of fusion, since the output feature maps f1, f2, f3, f4 have different scales of features, so f2, f3, f4 are up-sampled to the scale of f1 for connection. However, after up-sampling, the feature is prone to the problem that the ratio of the receptive field of the feature map and the text feature does not match, so we propose an adaptive fusion module to generate the feature attention map, and assign weights to each channel adaptively. The feature map of the channel is adaptively enhanced according to the size of the text instance. As shown in Fig (7), our module is augmented by attention to pixels rather than the correlation of global or local information, and we use residual connections. The AFM module is a differentiable module and secondly can be trained in an end-to-end manner.

First, the overall structure of the AFM module can be expressed as:

$$\begin{array}{ccc} F & = & f1\;\text{cat}\;f2↑f3↑f4↑ \end{array}$$ (14)

$$\begin{array}{ccc} F^{\prime } & = & F+F\cdot F_{\omega } \end{array}$$ (15)

$$\begin{array}{ccc} F_{\omega } & = & \text{softmax}(\text{BN}(\text{Conv1}(F))) \end{array}$$ (16)

We designed F_*ω*_ is the attention feature probability map output by the attention module. The essence of this module is to channel attention. Each channel’s pixel feature map is multiplied by different attention weights, where ⊙ means dot product, ⊕ means residual connection, and the weighted attention feature map is summed with the original feature map. The purpose of using pixel-by-pixel multiplication is to add attention to the channel of the feature, pay attention to the channel information, and multiply it with the original pixel map to better fuse the features and reduce the loss of information. In the gradient descent process, in order to prevent the gradient from disappearing, a corrected residual network is used.

For channel attention, we are different. Channel attention is realized by multiplying channels, modeling the importance of each feature channel, and then enhancing or suppressing it. Moreover, our fusion module is pixel attention, which achieves channel attention and focuses on pixel diversity in the same space. Our work can be expressed as follows:

$$\begin{array}{ccc} f^{\prime }\left(i,j,k\right) & = & f(i,j,k)+\omega (i,j,k)\cdot f(i,j,k) \end{array}$$ (17)

Where i, j, k represents the i row and j column in the kth channel, *ω* which represents the weight, and the weight value is adaptively assigned to the pixels of each channel to obtain the final weight probability distribution feature.

## Experiment

### Datasets

This paper conducts experiments on four open challenge datasets, namely CTW1500 [35], Total-Text [36], MARA-TD500 [5] and ICDAR2015 [37].

CTW1500 [35] is mainly used to detect text of arbitrary shape, including 1000 training pictures and 500 test pictures. It is a curved text dataset, mainly including English and Chinese. A single text line uses 32 numbers to annotate the position of the text box. The first four numbers are the coordinates of the rectangular text box, and the remaining 28 values represent 14 points that form a closed curved polygon text box.

Total-Text [36] is a scene text dataset in English. The dataset contains text instances of horizontal, multi-directional, and curved texts. The dataset is word-level annotated and contains 1255 training images and 300 testing images.

MSRA-TD500 [5] is an arbitrary directional scene text dataset consisting of out-door street scenes and indoor scenes such as offices and shopping malls, with indoor scenes mainly consisting of signs and door signs and outdoor scenes mainly consisting of billboards and warning signs. The dataset contains 300 training sets and 200 test sets, including Chinese and English languages. It is the annotation of line-level text, and the annotation is an external rotating rectangle of text lines.

ICDAR2015 [37] is one of the most commonly used scene text datasets. It has 1000 training pictures and 500 test pictures, which are mainly used for multi-directional text detection. It uses bounding box annotation, each line represents a text object, and a single object uses four points to represent the position information of the rectangular text box.

### Performance evaluation criteria

Three important metrics for general detection are recall (R), precision (P), and comprehensive metrics F-measure (F). The recall rate is the proportion of the entire sample predicted to be positive samples to all actual positive samples. The accuracy rate is the proportion of values that are correctly predicted as positive samples out of the predicted positive samples. The F-measure is the harmonic mean of the precision rate and the recall rate, and the F value is generally used to measure the quality of the overall algorithm.

### Implementation details

All experiments in this paper did not use external datasets pre-trained to finetune the network. Python 3.6 was used as the programming language in the framework of torch 1.6. All our experiments are performed on a 2080Ti under one GPU, each with a batch size of 4, trained from scratch on a public dataset for 600 epochs, similar to the PAN [7] algorithm, with an initial learning rate set to 1 × 10 − 3, use “poly” learning rate and “Adam” as optimizer. The dataset is augmented with data before being fed into the model, randomly scaling the images by a ratio of 0.7, 0.8, 0.9, 1.0, 1.1, 1.2, 1.3, rotating, flipping horizontally, and cropping the images. The image input size of ICDAR2015 and MSRA-TD500 is set to 736 × 736, and the input size of CTW1500 and Total-Text is 640 × 640.

### Ablation study

To better demonstrate the effectiveness of our module, an ablation study is performed on the arbitrary-shape text dataset CTW1500, which demonstrates the effectiveness of our cross-scale attention aggregation FPN network and adaptive fusion module.

In the ablation experiments, we test the effectiveness of the cross-scale attention module and the adaptive module on the dataset, respectively, and the detailed results are shown in Table [1]. In addition, we test the performance of our model on images of different scales, and the specific results are shown in Table [2]. We also tested the effectiveness of each component in CSAA-FPN, and the results are shown in Table [3].

**Table 1:** The ablation experiment on CTW1500. “P”, “R” and “F” respectively stand for “precision”, “recall” and “F-measure”, “N” means that the module is not used, and “Y” means that the module is used.

BackBone	CSAA-FPN	AFM	P(%)	R(%)	F(%)
ResNet-18	N	N	84.6	77.7	81.0
ResNet-18	Y	N	87.39	78.81	82.88
ResNet-18	N	Y	85.73	79.11	82.89
ResNet-18	Y	Y	86.17	80.25	83.11

**Table 2:** The results of our experiments at different scales on the CTW1500 dataset.

Method	P(%)	R(%)	F(%)
PAN-320	82.2	72.6	77.1
PAN-520	83.8	77.1	80.3
PAN-640	84.6	77.7	81.0
Our-320	84.97	72.78	78.41
Our-520	86.82	78.39	82.39
Our-640	86.17	80.25	83.11

**Table 3:** The results of CSAA-FPN with different settings the CTW1500 dataset. “concat” means adding only multi-scale concatenation to the CSAA-FPN, and “CBAM” means adding only the attention mechanism to the CSAA-FPN.

Method	P(%)	R(%)	F(%)
FPEM	84.6	77.7	81.0
concat	86.19	77.74	81.75
CBAM	87.01	78.13	82.33
CSAA-FPN	87.39	78.81	82.88

The effectiveness of our model can be seen in Table [1]. The precision, recall, and F-measure of the baseline model PAN on the public dataset CTW1500 are 84.6%, 77.7%, and 81.0%, respectively. Our method is also 86.17%, 80.25%, and 83.11% for precision, recall, and F-measure on this dataset. It can be seen that the accuracy of the method in this paper is 2.17% higher than the baseline model, the recall rate is 2.55% higher, and the F-measure is 2.11% higher. The detection results of this paper are significantly better than the PAN baseline network. We verify the performance of cross-scale attention aggregation FPN and adaptive fusion modules through ablation experiments. In the case of the CSAA-FPN+PAN, the entire model is 2.79% more accurate than our base-line PAN on CTW1500, the recall rate is improved by 1.11%, and the F-measure is improved by 1.88%. In the case of AFM+PAN, we achieve 1.13% higher precision, 1.41% higher recall, and 1.29% higher F-measure.

#### CSAA-FPN

CSAA-FPN is able to detect small text areas of arbitrary-shaped in complex contexts through constant fusion and supplementation when performing top-down and top-up path augmentation. We design two groups of experiments to verify the effectiveness of CSAA-FPN. Firstly, we make a comparison between the model with CSAA-FPN and without CSAA-FPN. It can be seen from Table [1], CSAA-FPN significantly improves the F-measure by 1.88% over the baseline. Secondly, we subdivided CSAA-FPN and the experimental results are shown in Table [3]. We can find that CSAA-FPN is the result of a combination of multi-scale aggregation and attention mechanisms. In CSAA-FPN, on the one hand, concatenation aggregation can fully fuse the features of multiple levels on the channel and make full use of each level’s features. Compared with the baseline, the F-measure after adding the concatenation operation is improved by 0.75%. On the other hand, the CBAM attention mechanism can effectively align semantic features and suppress redundant information of complex backgrounds. Compared with the baseline and model, the F-measure value after adding the attention mechanism is improved by 1.22%. These performance enhancements indicate that CSAA-FPN can indeed extract feature information more comprehensively and improve the accuracy of image classification. Compared to the original Feature Pyramid Enhancement Module (FPEM), it has stronger feature capture capability, higher detection ability for small objects, and more accurate separation of adjacent texts.

#### AFM

AFM weights the output features, which can effectively enhance useful information, suppress worthless information, and make the prediction box closer to the real box. As shown in Table [1], it boosts the baseline by 1.29%. By adding AFM to the model equipped with both CSAA-FPN and PAN, an additional gain of 0.22% can be achieved. Experimental results show that the detection performance of the network combining the advantages of these two modules is better than that of a single module applied separately.

### Compare with previous method

To further verify the robustness of our model, we compare with previous state-of-the-art methods on arbitrary-shaped text datasets, curved text datasets, multi-lingual text datasets, and long-text scene datasets, respectively. The experimental results are shown in Tables [4–7]. Compared with the baseline network PAN, the method in this paper has obvious improvement in each dataset, especially on the multilingual text dataset, which has the best effect and the best performance. Also relative to other methods, the method in this paper also shows state-of-the-art performance.

**Table 4:** Results at 640 × 640 on CTW1500, pre-train represents whether to use the pre-training dataset for fine-tuning. “Y” means using pre-training, “N” means not using pre-training. “P”, “R” and “F” stand for “precision”, “recall” and “F-measure” respectively.* indicates the results from [7]. PAN++ [8] is the detection module.

Method	Pre-train	P(%)	R(%)	F(%)
CTPN* [38]	N	60.4	53.8	56.9
SegLink* [13]	N	42.3	40.0	40.8
EAST* [19]	N	78.7	49.1	60.4
Textsnake [23]	Y	67.9	85.3	75.6
CTD+TLOC [41]	N	77.4	69.8	73.4
PSENet-1s [28]	N	80.6	75.6	78.0
PSENet-1s [28]	Y	84.8	79.7	82.2
Textfield [42]	Y	83.0	79.8	81.4
CRAFT [27]	Y	86.0	81.1	83.5
Jiang et al. [39]	Y	84.9	80.3	82.5
Qin et al. [40]	N	81.8	76.8	79.4
DB-ResNet18. [44]	Y	84.8	77.5	81.0
PAN-640 [7]	N	84.6	77.7	81.0
PAN++ [8]	N	86.52	79.27	82.74
Ours	N	86.17	80.25	83.11

**Table 5:** Results at 640 × 640 on total-text dataset, pre-train represents whether to use the pre-training dataset for fine-tuning. “Y” means using pre-training, “N” means not using pre-training. “P”, “R” and “F” stand for “precision”, “recall” and “F-measure” respectively. * indicates the results from [7]. PAN++ [8] is the detection module.

Method	Pre-train	P(%)	R(%)	F(%)
SegLink* [13]	Y	30.3	23.8	26.7
EAST* [19]	N	50.0	36.2	42.0
Deconvnet [36]	N	33.0	40.0	36.0
Textsnake [23]	N	82.7	74.5	78.4
PSENet-1s [28]	N	81.8	75.1	78.3
PSENet-1s [28]	Y	84.0	78.0	82.4
SPCNet [20]	Y	83.0	82.8	82.9
CRAFT [27]	Y	87.6	79.6	83.6
Textfield [42]	Y	81.2	79.9	80.6
LOMO [43]	Y	87.6	79.3	83.3
DB-ResNet18(800 × 800) [44]	Y	88.3	77.9	82.8
PAN-640 [7]	N	87.15	79.5	83.15
PAN++ [8]	N	87.86	79.12	83.26
Ours	N	88.48	78.81	83.92

**Table 6:** Experimental results at MSRA-TD500 with a scale of 736 × 736, pre-train represents whether to use the pre-training dataset for fine-tuning. “Y” means using pre-training, “N” means not using pre-training. “P”, “R” and “F” stand for “precision”, “recall” and “F-measure” respectively. PAN++ [8] is the detection module.

Method	Pre-train	P(%)	R(%)	F(%)
EAST [19]	N	87.3	67.3	76.1
DeepReg [46]	N	77.0	70.0	74.0
SegLink [13]	Y	86.0	70.0	77.0
PixeLink [25]	Y	83.0	73.2	77.8
RRD [15]	Y	87.0	73.0	79.0
MCN [45]	Y	88.0	79.0	83.0
RRPN [11]	N	82.0	68.0	74.0
TextSnake [23]	Y	83.2	73.9	78.3
CRAFT [27]	Y	88.2	78.2	82.9
DB-ResNet18(736 × 736) [44]	Y	90.4	76.3	82.8
PAN-640 [7]	N	80.7	77.3	78.6
PAN++ [8]	N	85.25	79.73	82.39
Ours	N	85.44	81.8	83.58

**Table 7:** Experimental results at dataset ICADR2015 with a scale of 736 × 736, pre-train represents whether to use the pre-training dataset for fine-tuning. “Y” means using pre-training, “N” means not using pre-training. “P”, “R” and “F” stand for “precision”, “recall” and “F-measure” respectively. PAN++ [8] is the detection module.

Method	Pre-train	P(%)	R(%)	F(%)
CTPN [38]	Y	74.2	51.6	60.9
EAST [19]	Y	83.6	73.5	78.2
DeepReg [18]	Y	82.0	80.0	81.0
SegLink [13]	N	73.1	76.8	75.0
WordSup [46]	N	79.3	77.0	78.2
RRPN [11]	Y	82.0	73.0	77.0
PixelLink [25]	N	82.9	81.7	82.3
PSE Net-1s [28]	N	79.7	81.5	80.6
Pelee-Text++ [47]	Y	76.7	87.5	81.7
Qin et al. [40]	N	80.2	82.86	81.56
Jiang et al. [39]	N	79.7	85.8	82.6
DB-ResNet18 [44]	Y	86.8	78.4	82.3
PAN [7]	N	82.9	77.8	80.3
PAN++ [8]	N	85.13	79.97	82.47
Ours	N	86.22	79.24	82.58

#### Curved text detection

**Fig 8:**
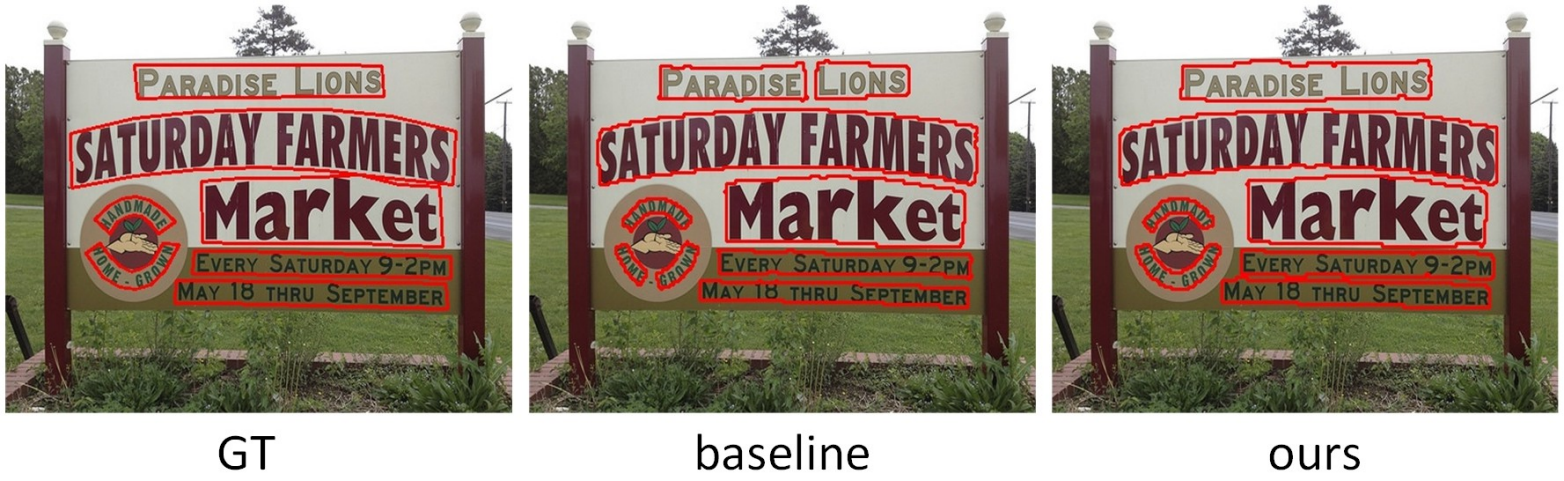
The visualization results of GT, baseline and our method on CTW1500.

**Fig 9:**
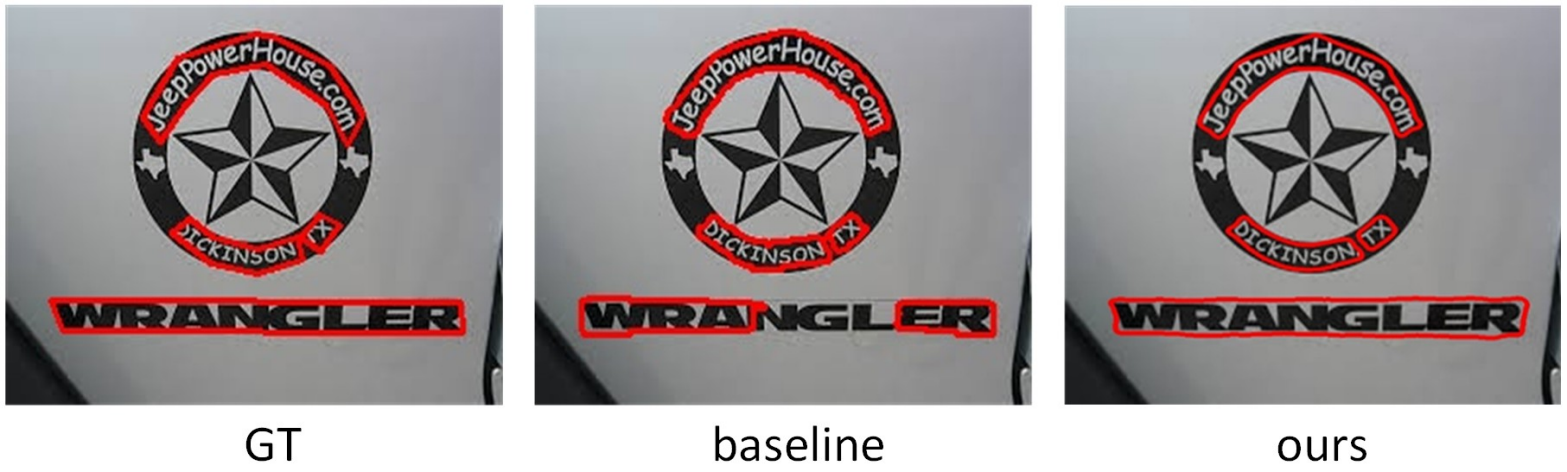
The visualization results of GT, baseline and our method on Total-Text.

To evaluate the performance of our model for detecting curved text instance, we compare our model with other state-of-the-art methods on CTW1500 and Total-Text. The visualization results of our model on the curved text datasets CTW1500 and Total-Text are shown in Figs (8) and (9).

The evaluation results on CTW1500 are shown in Table [4], the methods of CTPN and SegLink are mainly for horizontal text detection, so they cannot detect curved text well. Compared with the previous pre-trained text bending detector TextSnake, the precision of this method without pre-training has increased by 18.27%, and the F-measure has increased by 7.51%. Textsnake has a complex post-processing process, which shows the superiority of our algorithm. Compared with CRAFT, the most superior algorithm in recent years, our algorithm is slightly lower than CRAFT without pretraining. The CRAFT algorithm is a character-level segmentation algorithm that links characters after passing through a single character, so the noise introduced is slight, the weakly supervised learning method is used, and the detection performance is better, but the post-processing algorithm of CRAFT is also more complicated. Therefore, the feature enhancement algorithm proposed in this paper combined with the post-processing algorithm of PAN has better performance.

To verify the model’s effectiveness on curved text, we also evaluate our model on the Total-Text dataset. As shown in Table [5], in the absence of any external dataset pre-training, the F-measure of the curved text detector Textsnake is improved by 5.52%. Compared with other advanced methods, the F-measure of our method without pre-training is better than the current state-of-the-art method after pre-training CRAFT is 0.3% higher, 1% higher than SPCNet, 2.63% higher than DB-Resnet18, and our F-measure improves by 0.77% compared to the baseline method. Because the Total-Text dataset consists primarily of large-scale curved text instances, whereas our model is focuses on detecting small target text and separating adjacent text target instances, so it does not significantly improve on this dataset. However, the proposed AAF-Net achieve F-measures of 83.92% when the external data is not used, which proves the effectiveness of our model for curved text.

Fig (8) shows the visualization results of GT, baseline and our method on the curved text dataset CTW1500, respectively. In Fig (8), it can be seen that the baseline model produces severe false detections in dense text regions, splitting one text instance into several shorter text instances, and failing to accurately detect curved text instances. However, our model makes full use of the contextual semantic information to accurately segment the regions in the graph. Fig (9) shows the visualization results on GT, baseline and our method on Total-Text, respectively. The baseline model not only misses the text instance in the last line of the image, but also incorrectly divides this proposal into two text proposals, and our model solves this problem well.

#### Multi-language text detection

**Fig 10:**
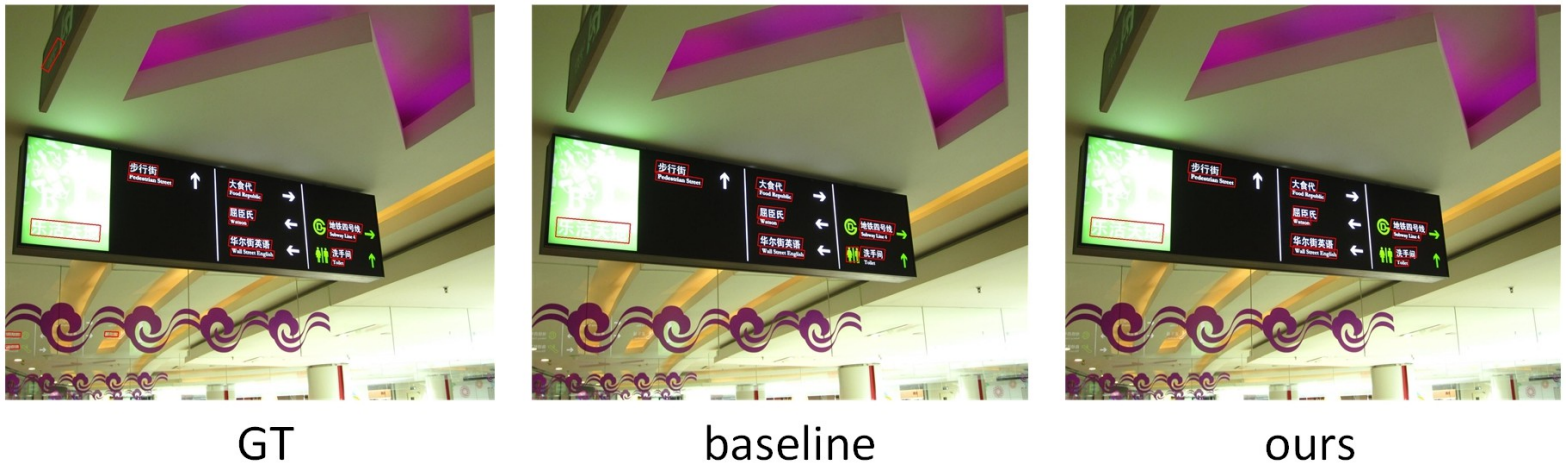
The visualization results of GT, baseline and our method on MSRA-TD500.

We conduct experiments on MSRA-TD500 to demonstrate the robustness of our method for detecting multilingual text instances. In Table [6], compared with the base-line algorithm, the precision, recall and F-measure are increased by 4.74%, 4.5% and 4.74%, respectively. Compared with the most classic algorithm CRAFT, F-measure are increased by 0.78%. To summarize, AAF-Net can achieve higher performance and can be deployed in complex natural scenarios. Fig (10) shows the visualization results MSRA-TD500.

The text instances in MSRA-TD500 contain many large and long texts, proving that our model also has superior performance on multilingual and lengthy text data. On the multilingual text dataset, the texts in different languages have different characteristics, and there are huge differences. For example, Chinese text is larger and has more complex fonts. In contrast, English text instances have smaller fonts, making our model more capable of quickly detecting text features at different scales. Hence, the robustness is better on this dataset and the enhancement is better.

Fig (10) shows the visualization results on GT, baseline and our method on the multilingual text dataset MSRA-TD500, respectively. From the visualization results, it can be seen that the baseline model did not detect the small target text on the sign, while our model detected all text instances on the sign. However, because the text in the upper and lower regions is too blurred, so our model does not fully detect all text instances. Although some text situations cannot be successfully detected in some extreme environments, our model shows robustness to small scale text instances. Compared with baseline models, AAF-Net exhibits its superior performance.

#### Multi-oriented text detection

The evaluation of our model on the ICDAR15 dataset is shown in Table [7], which mainly includes horizontal, vertical and oblique scene texts. Our method achieves 86.22%, 79.24% and 82.58% in the precision, recall and F-measure without pre-training, and the F-measure is 2.28% higher than the baseline algorithm. Compared with other representative methods, it also has superior performance. For example, compared with RRPN, P, R, and F are improved by 6.22%, 6.24%, and 5.58%, respectively. Compared with EAST, its F-measure is improved by 4.38%. Our method also achieves significant improvement over the state-of-the-art model DB-ResNet18, demonstrating the robustness of our model on this dataset.

**Fig 11:**
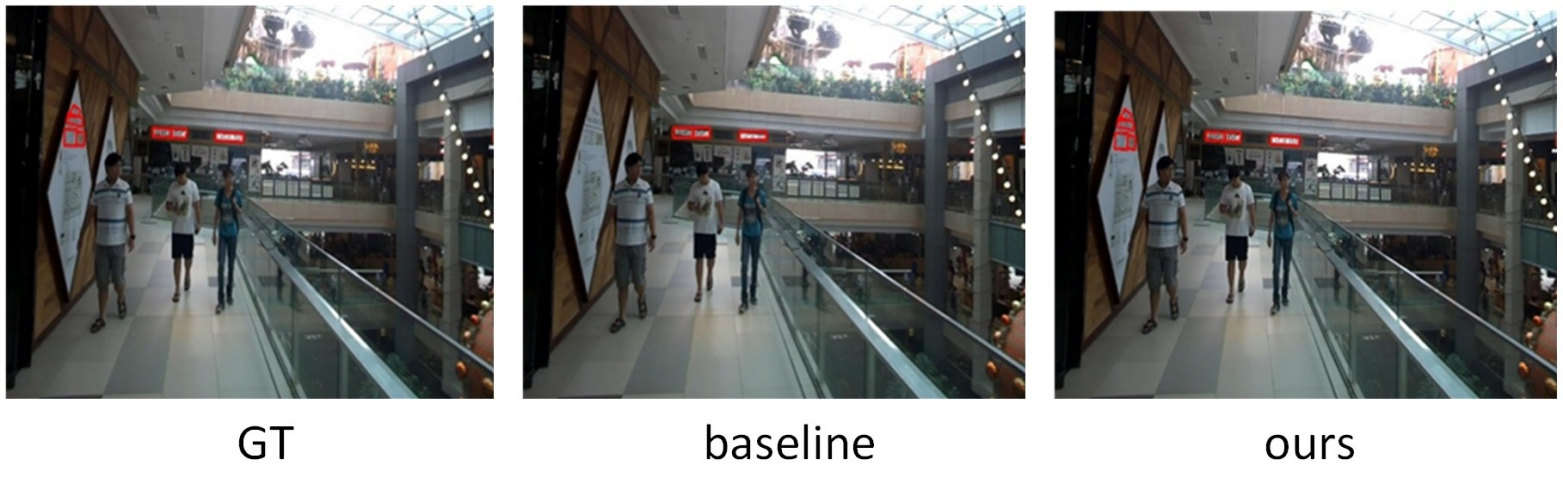
The visualization results of GT, baseline and our method on ICDAR2015.

Fig (11) shows the visualization results of GT, baseline and our method, respectively. Because the font is too small in Fig (11), the baseline model fails to detect all text instances. However, our cross-scale attention aggregation module better integrates multi-scale information, and it is able to detect different scale text instances and adjacent text. So, our model is able to fully detect all text instances.

## Discussion

From the visualization results of the experiment, the PAN [7] algorithm has serious false detection and missed detection in the detection of adjacent text, and our model can effectively solve this problem. Because the FPEM in the PAN algorithm ignores the semantic difference between feature maps due to different depths in the process of path enhancement, it is easy to cause aliasing effects and cannot fully utilize the semantic information. While our CSAA-FPN network is designed to enhance the ability of feature pyramid representation and aggregate textual information, the attention mechanism can better align semantic information, enhance contextual connections, identify background information, and eliminate aliasing effects. So, our network can separate adjacent text and detect small target text instances more accurately than the original algorithm. Coupled with our AFM module, which pays attention to the channel information of the output features, this further refines the features and pays more attention to the pixel information of the output features. Therefore, our network has efficient detection ability and certain robustness.

## Conclusion

Aiming at the problem that existing lightweight scene text detection methods cannot accurately detect small target text instances in any direction, and the detection rate of mutually adhering text instances is low, we propose a detection framework that can detect arbitrary text shapes. The network is divided into three parts: feature extraction, feature enhancement, and post-processing algorithm. The cross-scale attention aggregation pyramid network enhances the extracted features and solves the problem of text detection at different scales, and we introduce the CBEM attention module to focus on crucial information, so that the output of each layer provides rich semantic and geometric information. In this paper, an end-to-end trainable lightweight network is designed to be applied in the final feature fusion to highlight the representation of information and obtain more accurate text features. Then, a post-processing algorithm of pixel aggregation is used to obtain a lightweight and efficient text detector of arbitrary shapes. Experiments on several public text datasets demonstrate the superior ad-vantages when compared to previous state-of-the-art text detectors.

Considering that our model cannot fully detect text instances in some severe environments, in the future, we hope to improve our accuracy further and be able to detect all text instances in any environment. Moreover, our model can be applied to a variety of detecting tasks. It should be noted that when we add attention to the network structure, a small number of parameters are inevitably introduced, resulting in a slight increase in the model parameters. So, we intend to improve the network post-processing method to make it lighter and more efficient. We also consider applying our algorithm to the task of Chinese language detection and recognition in the future.
